# Boosting mitochondria activity by silencing MCJ overcomes cholestasis-induced liver injury

**DOI:** 10.1016/j.jhepr.2021.100276

**Published:** 2021-03-18

**Authors:** Paula Iruzubieta, Naroa Goikoetxea-Usandizaga, Lucía Barbier-Torres, Marina Serrano-Maciá, David Fernández-Ramos, Pablo Fernández-Tussy, Virginia Gutiérrez-de-Juan, Sofia Lachiondo-Ortega, Jorge Simon, Miren Bravo, Fernando Lopitz-Otsoa, Mercedes Robles, Carlos Ferre-Aracil, Marta Varela-Rey, Natalia Elguezabal, José Luis Calleja, Shelly C. Lu, Malgorzata Milkiewicz, Piotr Milkiewicz, Juan Anguita, María J. Monte, José J.G. Marin, Marcos López-Hoyos, Teresa C. Delgado, Mercedes Rincón, Javier Crespo, María Luz Martínez-Chantar

**Affiliations:** 1 Gastroenterology and Hepatology Department, Marqués de Valdecilla University Hospital, Clinical and Translational Digestive Research Group, IDIVAL, Santander, Spain; 2 Liver Disease and Liver Metabolism Laboratory, CIC bioGUNE-BRTA (Basque Research & Technology Alliance), Centro de Investigación Biomédica en Red de Enfermedades Hepáticas y Digestivas (CIBERehd), Derio, Bizkaia, Spain; 3 Liver Unit, Vírgen de Victoria University Hospital, Gastroenterology Service and Department of Medicine, University of Málaga, Malaga, Spain; 4 Liver Unit, Puerta de Hierro University Hospital, IDIPHISA, CIBERehd, Madrid, Spain; 5 Departmento de Sanidad Animal, NEIKER-Instituto Vasco de Investigación y Desarrollo Agrario, Derio, Spain; 6 Division of Digestive and Liver Diseases, Cedars-Sinai Medical Center, Los Angeles, CA, USA; 7 Department of Medical Biology, Pomeranian Medical University, Szczecin, Poland; 8 Liver and Internal Medicine Unit, Medical University of Warsaw, Warsaw, Poland; 9 Inflammation and Macrophage Plasticity Laboratory, CIC bioGUNE-BRTA (Basque Research & Technology Alliance), Derio, Spain; 10 Ikerbasque, Basque Foundation for Science, Bilbao, Spain; 11 Experimental Hepatology and Drug Targeting (HEVEFARM), IBSAL, University of Salamanca, Salamanca, Spain; 12 Immunology Department, University Hospital Marqués de Valdecilla, IDIVAL, Santander, Spain; 13 Department of Medicine, University of Vermont College of Medicine, Burlington, VT, USA

**Keywords:** Cholestasis, Mitochondria, MCJ, ROS, Bile duct ligation, α-SMA, alpha-smooth muscle actin, Abs, antibodies, ALP, alkaline phosphatase, ALT, alanine aminotransferase, AMA-M2, antimitochondrial M2 antibody, ANA, antinuclear antibodies, APRI, AST to platelet ratio index, AST, aspartate aminotransferase, BA, bile acid, BAX, BCL2 associated X, BCL-2, B-cell lymphoma 2, BCL-Xl, B-cell lymphoma-extra large, BDL, bile duct ligation, Ccl2, C-C motif chemokine ligand 2, Ccr2, C-C motif chemokine receptor 2, Ccr5, C-C motif chemokine receptor 5, CLD, cholestatic liver disease, Cxcl1, C-X-C motif chemokine ligand 1, Cyp7α1, cholesterol 7 alpha-hydroxylase, DCA, deoxycholic acid, ETC, electron transport chain, Ezh2, enhancer of zeste homolog 2, Fxr, farnesoid X receptor, GAPDH, glyceraldehyde-3-phosphate dehydrogenase, GCDCA, glycochenodeoxycholic acid, Hif-1α, hypoxia-inducible factor 1-alpha, HSC, hepatic stellate cells, JNK, c-Jun N-terminal kinase, KO, knockout, LSM, liver stiffness, MAPK, mitogen-activated protein kinase, mRNA, messenger ribonucleic acid, MCJ, methylation-controlled J, MLKL, mixed-lineage kinase domain-like pseudokinase, MMP, mitochondrial membrane potential, MPO, myeloperoxidase, MPT, mitochondrial permeability transition, Nrf1, nuclear respiratory factor 1, PARP, poly (ADP-ribose) polymerase, PBC, primary biliary cholangitis, Pgc1α, peroxisome proliferator-activated receptor gamma coactivator 1-alpha, Pgc1β, peroxisome proliferator-activated receptor gamma coactivator 1-beta, p-JNK, phosphor-JNK, p-MLKL, phosphor-MLKL, PSC, primary sclerosing cholangitis, ROS, reactive oxygen species, RT, room temperature, SDH_2_, succinate dehydrogenase, shRNA, small hairpin RNA, siRNA, small interfering RNA, tBIL, total bilirubin, Tfam, transcription factor A mitochondrial, TNF, tumour necrosis factor, Trail, TNF-related apoptosis-inducing ligand, Ucp2, uncoupling protein 2, UDCA, ursodeoxycholic acid, VCTE, vibration-controlled transient elastography, WT, wild-type

## Abstract

**Background & Aims:**

Mitochondria are the major organelles for the formation of reactive oxygen species (ROS) in the cell, and mitochondrial dysfunction has been described as a key factor in the pathogenesis of cholestatic liver disease. The methylation-controlled J-protein (MCJ) is a mitochondrial protein that interacts with and represses the function of complex I of the electron transport chain. The relevance of MCJ in the pathology of cholestasis has not yet been explored.

**Methods:**

We studied the relationship between MCJ and cholestasis-induced liver injury in liver biopsies from patients with chronic cholestatic liver diseases, and in livers and primary hepatocytes obtained from WT and MCJ-KO mice. Bile duct ligation (BDL) was used as an animal model of cholestasis, and primary hepatocytes were treated with toxic doses of bile acids. We evaluated the effect of MCJ silencing for the treatment of cholestasis-induced liver injury.

**Results:**

Elevated levels of MCJ were detected in the liver tissue of patients with chronic cholestatic liver disease when compared with normal liver tissue. Likewise, in mouse models, the hepatic levels of MCJ were increased. After BDL, MCJ-KO animals showed significantly decreased inflammation and apoptosis. In an *in vitro* model of bile-acid induced toxicity, we observed that the loss of MCJ protected mouse primary hepatocytes from bile acid-induced mitochondrial ROS overproduction and ATP depletion, enabling higher cell viability. Finally, the *in vivo* inhibition of the MCJ expression, following BDL, showed reduced liver injury and a mitigation of the main cholestatic characteristics.

**Conclusions:**

We demonstrated that MCJ is involved in the progression of cholestatic liver injury, and our results identified MCJ as a potential therapeutic target to mitigate the liver injury caused by cholestasis.

**Lay summary:**

In this study, we examine the effect of mitochondrial respiratory chain inhibition by MCJ on bile acid-induced liver toxicity. The loss of MCJ protects hepatocytes against apoptosis, mitochondrial ROS overproduction, and ATP depletion as a result of bile acid toxicity. Our results identify MCJ as a potential therapeutic target to mitigate liver injury in cholestatic liver diseases.

## Highlights

• Hepatic MCJ levels were upregulated in patients with cholestatic liver disease.
• MCJ absence resulted in decreased liver inflammation, neutrophil activation, and mitochondrial dysfunction after BDL.
• MCJ deficiency protected the hepatocytes from ROS overproduction and ATP depletion.
• *In vivo* inhibition of MCJ expression mitigated liver injury by bile acids.
• Our results identified MCJ as a key regulator of cholestatic liver disease.

## Graphical abstract

**Figure:**
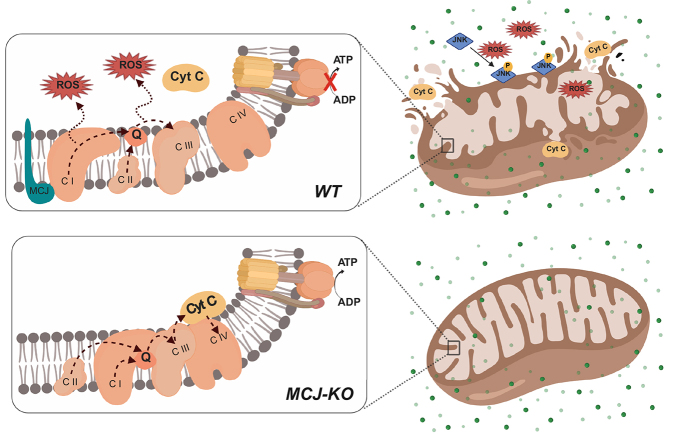

## Introduction

Cholestasis, defined as any condition that causes the retention and accumulation of potentially toxic bile acids (BAs) in the liver and plasma, can be triggered by different causes, such as toxins, infection, inflammation, autoimmune diseases, or obstruction of the biliary tree. The most common chronic cholestatic liver diseases (CLDs) in adults are primary biliary cholangitis (PBC) and primary sclerosing cholangitis (PSC), which are characterised by chronic bile duct inflammation that leads to cholestasis.^1^ Both diseases can eventually progress to liver cirrhosis, liver failure, and death despite the currently available drug treatments.^2^

Hepatic inflammation is an important feature of CLD in both humans and experimental animals.[3], [4], [5] Cholestasis can be reproduced in rodents by surgical ligation of the common bile duct.^6^ After bile duct ligation (BDL) in mice, Kupffer cells are activated and other inflammatory cells, particularly neutrophils, accumulate in the liver causing cell injury mainly through the formation of reactive oxygen species (ROS) and the release of proteases.^5^^,^^7^

The inappropriate activation of programmed death, apoptosis, and necroptosis has been associated with the pathogenesis of CLD.^8^^,^^9^ Apoptosis and necroptosis can be executed upon ligation of the death receptor subset of the tumour necrosis factor (TNF) receptor family. However, apoptosis has long been recognised as a direct consequence of BA-mediated injury.^8^ In this sense, a variety of key events in apoptosis focus on the mitochondria. In fact, several studies indicated that mitochondrial dysfunction and oxidative stress are the main mechanisms of cholestasis-induced liver injury.[10], [11], [12]

Mitochondria are the main energy source in hepatocytes and play a major role in the oxidative metabolism and normal function of the liver. The transport of electrons by the different complexes of the electron transport chain (ETC) and the generation of mitochondrial membrane potential (MMP) are key for ATP synthesis. Some electrons may escape and lead to ROS generation.^13^^,^^14^

A reduction in general mitochondrial function has been observed during experimental cholestasis, which was associated with a decrease in the oxidative metabolism and ATP synthesis as well as ROS overproduction.^15^^,^^16^ BAs are amphipathic molecules and may interact with cell membranes and proteins, interfering with their function. Thus, several mechanisms involved in mitochondrial dysfunction caused by BAs have been described: the altered activity of different ETC complexes,^10^ modified mitochondrial membrane permeability,^11^^,^^17^ and decreased mitochondrial biogenesis.^18^^,^^19^

Mitochondrial respiration is regulated by the metabolic needs of the cells and it can be modulated in response to acute or chronic metabolic changes. An endogenous negative regulator of MMP and ATP synthesis was identified a few years ago – methylation-controlled J protein (MCJ).^20^ MCJ, also known as DnaJC15, is a transmembrane protein that is found in the inner mitochondrial membrane, whose absence leads to increased complex I activity and mitochondrial respiration in breast cancer, immune cells, and the liver.[20], [21], [22]

A lack of MCJ stimulates the formation of respiratory supercomplexes facilitating electron transfer between complexes and minimising their leakage, which leads to lower ROS production.^20^ Although under normal physiological conditions MCJ is dispensable, MCJ deficiency in mice prevents liver injury in response to lipid accumulation^23^ and known hepatotoxins, such as lipopolysaccharide and acetyl-para-aminophenol,^22^^,^^24^ suggesting that the regulation of the mitochondrial activity by MCJ modulates the response to liver toxicants.

In this study, we investigated the role of MCJ in the pathogenesis of CLD. We show that the hepatic MCJ levels were upregulated in patients with CLD and in mice after BDL. The lack of MCJ in a murine BDL model reduced the neutrophil activation and prevented mitochondrial dysfunction, thus ameliorating cholestatic liver injury. In addition, the loss of MCJ protected hepatocytes from mitochondrial ROS overproduction and ATP depletion as a result of BA toxicity, which led to decreased BA-induced hepatocyte death. Therefore, our results identify MCJ as a key regulator of CLD and a potential therapeutic target to mitigate cholestasis-induced liver injury.

## Materials and methods

### Human samples

A total of 267 patients with CLD, PBC (n = 197), and PSC (n = 70), were included in this study, 205 of them with serum samples, 37 with paraffin-embedded liver biopsies, and 25 with frozen liver tissue samples. The characteristics of these patients are summarised in Table 1, Table 2. The diagnosis of PBC and PSC, established at the Marqués de Valdecilla Hospital (Santander, Spain), Pomeranian Medical University (Szczecin, Poland), Puerta de Hierro Hospital (Madrid, Spain), and Virgen de Victoria Hospital (Málaga, Spain), was based on clinical and biochemical data, immunological markers, imaging, features of liver histology, and the exclusion of other possible causes of liver injury. The disease stage was defined as early or advanced disease according to EASL clinical practice guidelines.^1^

**Table 1:** Characteristics of patients with biopsy-proven cholestatic liver disease.

	All patients n = 62	Early disease n = 34	Advanced disease n = 28	*p* value∗
Sex (F/M)	50/12	28/6	22/6	0.71
Aetiology (PBC/PSC)	50/12	30/4	20/8	0.12
Age (years)	48.8 ± 12.0	48.2 ± 9.8	49.6 ± 14.4	0.68
AMA+ (%)	82.6	88.9	60	0.19
ANA+ (%)	17.4	11.1	40	0.19
AST (U/L)	75.9 ± 48.3	52.9 ± 23.5	111.2 ± 55.5	**<0.01**
ALT (U/L)	86.8 ± 62.0	80.3 ± 43.2	95.1 ± 80.6	0.49
GGT (U/L)	237.4 ± 341.1	214.0 ± 173.9	267.3 ± 482.6	0.22
ALP (U/L)	315.9 ± 356.7	239.5 ± 234.3	432.4 ± 472.2	**<0.01**
tBIL (mg/dl)	3.7 ± 6.2	1.0 ± 1.7	7.7 ± 8.2	**<0.01**
Albumin (g/dl)	3.9 ± 0.7	4.3 ± 0.4	3.4 ± 0.6	**<0.01**
Platelets (10^9^/L)	212.9 ± 100.9	262.3 ± 78.6	149.8 ± 92.0	**<0.01**
MCJ (% positive area)	49.9 ± 22.0	51.6 ± 22.8	46.7 ± 20.8	0.52

Data are expressed as mean ± SD. ∗Early disease *vs.* advanced disease (Chi-square or Student *t* or Mann-Whitney *U* test). Bold values denote statistical significance. ALP, alkaline phosphatases; ALT, alanine aminotransferase; AMA, antimitochondrial antibodies; ANA, antinuclear antibodies; AST, aspartate aminotransferase; GGT, gamma-glutamyl transpeptidase; MCJ, methylation-controlled J; tBIL, total bilirubin.

**Table 2:** Characteristics of cholestatic liver disease-diagnosed patients with serum samples.

	All patients n = 205	Early disease n = 152	Advanced disease n = 53	*p* value∗
Sex (F/M)	155/50	116/36	39/14	0.69
Aetiology (PBC/PSC)	147/58	108/44	39/14	0.72
Age (years)	51.4 ± 15.3	50.9 ± 15.6	53.0 ± 14.3	0.40
AMA+ (%)	55.5	56.4	52.9	0.67
ANA+ (%)	37.2	33.1	49.0	**0.04**
IgM (mg/dl)	238.8 ± 157.4	224.1 ± 159.2	281.4 ± 144.4	**<0.01**
AST (U/L)	52.2 ± 67.0	45.6 ± 71.4	70.4 ± 48.9	**<0.01**
ALP (U/L)	190.5 ± 161.4	171.6 ± 132.0	244.8 ± 218.1	**<0.01**
tBIL (mg/dl)	1.3 ± 2.1	1.0 ± 1.6	2.3 ± 2.8	**<0.01**
Albumin (g/dl)	4.2 ± 0.6	4.4 ± 0.4	3.7 ± 0.7	**<0.01**
Platelet (10^9^/L)	210.8 ± 88.0	246.0 ± 58.8	109.7 ± 79.7	**<0.01**
LSM by VCTE (kPa)	8.5 ± 7.9	5.7 ± 1.6	20.8 ± 11.8	**<0.01**
APRI	0.9 ± 1.4	0.5 ± 0.8	2.2 ± 1.7	**<0.01**
Inadequate response to UDCA (%)^#^	50.2	39.5	81.1	**<0.01**
Anti-MCJ Ab (%)	17.1	15.8	20.8	0.41

^#^Biochemical response to UDCA was assessed using Paris-II criteria. Data are expressed as mean ± SD. ∗Early disease *vs.* advanced disease (Chi-square or Student *t* or Mann-Whitney *U* test). Bold values denote statistical significance. Ab, antibody; ALP, alkaline phosphatases; AMA, antimitochondrial antibodies; ANA, antinuclear antibodies; APRI, AST to platelet ratio index; AST, aspartate aminotransferase; LSM, liver stiffness; tBIL, total bilirubin; UDCA, ursodeoxycholic acid; VCTE, vibration-controlled transient elastography.

Healthy human liver samples (n = 25; 52% women, mean age 62.1 years, 29–81 years), 18 of them with paraffin-embedded liver biopsies, and 7 with frozen liver tissue samples, from organ donor subjects from Marqués de Valdecilla Hospital and Pomeranian Medical University; and serum samples (n = 36; 77.8% women, mean age 54.5 years, 27–68 years) from healthy subjects provided by BioBank Valdecilla (PT17/0015/0019) were used as controls.

The study was performed in agreement with the Declaration of Helsinki, and with local and national laws. The Cantabria’s Research Ethics Committee approved the study procedures (code 2017.052) and written informed consent was obtained from all patients.

### Experimental procedures in animals

MCJ-knockout (KO) animals were generated as previously described.^20^ MCJ-KO mice and wild-type (WT) mice were in the C57Bl/6J background. The MCJ KO mice were backcrossed over 10 times. The lines were derived from heterozygous founders. Both colonies were bred at the CIC bioGUNE animal facility. All animal procedures were approved following European and Spanish regulations. The CIC bioGUNE Animal Facility is an AALAC Intl. accredited facility.

Three-month-old male WT and MCJ-KO mice were subjected to complete BDL as described.^25^ Liver and serum samples were harvested, snap frozen in liquid nitrogen, and stored at -80^o^C for subsequent analysis.

### *In vivo* silencing

For MCJ silencing, WT mice were subjected to BDL, and 3 and 5 days after the surgery, the animals were divided into 2 groups and received either 200 μl of a 0.75-μg/μl solution of MCJ-specific siRNA (si*Mcj*) (n = 5) or control siRNA (siControl) (n = 6) using Invivofectamine® 3.0 Reagent (Thermo Fisher Scientific, USA) through tail vein injection. The animals were sacrificed 7 days after BDL.

### Histology

Paraffin-embedded liver samples were sectioned, dewaxed, and hydrated. All procedures were performed according to standard protocols using the EnVision+System HRP (Dako, Denmark). The samples were incubated with Vector Vip substrate (Vectorlabs, Burlingame, USA) for colour development. About 5–10 random images per sample were taken with a ×20 or x10 objective from an AXIO Imager A1 microscope (Carl Zeiss AG, Jena, Germany). Quantification of the staining intensity, average sum of intensities, and stained area percentage of each sample were calculated using FRIDA software http://bui3.win.ad.jhu.edu/frida/ (Johns Hopkins University). Histological techniques are detailed in the Supplementary data.

### ELISA for anti-MCJ antibodies

The presence of anti-MCJ antibodies (Abs) in serum was measured with an in-house ELISA method. Briefly, microtitration plates were coated overnight at room temperature (RT) with 100 μl of human N terminus peptide of MCJ per well diluted in borate-buffered saline to a final concentration of 8 μg/ml. After washing, 250 μl of blocking buffer (2% bovine serum albumin in PBS) was added to each well and incubated for 1 h at RT. Thereafter, 100 μl of serum from patients or controls, diluted at 1:25 in blocking buffer, was added to each well and incubated at RT for 2 h.

Afterward, the plates were washed and polyclonal goat anti-human IgG conjugated to alkaline phosphatase (Dako, Gloostrup, Denmark), diluted 1:500 in blocking buffer, was added to each well and incubated for 2 h at RT. After 3 additional washes, the presence of anti-MCJ Abs was determined following a 3-h incubation with p-nitrophenyl phosphate disodium (Sigma-Aldrich). The values were obtained as absorbance units at 405 nm. A result was considered positive when its value exceeded that of the average of the healthy control population values by at least 3 SDs.

### Liver transaminase activity determination in mouse serum

The alanine aminotransferase (ALT) serum activity was determined using the Selectra Junior Spinlab 100 automated analyser (Vital Scientific) according to the manufacturer’s instructions. Standard controls were run before each determination.

### BA measurement

The total BA levels in the serum and liver tissue were determined using Total Bile Acid Assay Kits (Cell Biolabs), following the recommendations of the manufacturer. Twenty-three BAs were separated and detected with HPLC-tandem mass spectrometry as previously described,^26^^,^^27^ and using a 6420 Triple Quad LC/MS device (Agilent Technologies).

### Mouse TNF quantification in serum

The tumour necrosis factor (TNF) levels were determined by ELISA using the DuoSet II kit (R&D Systems) according to the manufacturer’s recommendations.

### Flow cytometry

Blood was extracted from the saphenous vein in the presence of EDTA and depleted of erythrocytes by hypotonic lysis. The cells (106/ml) were then incubated with Fc Block (anti-CD16/CD32; BD BioSciences) and tagged with fluorochrome-labelled antibodies against GR-1, (Miltenyi Biotec).

### Myeloperoxidase activity

Myeloperoxidase activity was measured in murine sera using the Myeloperoxidase Colorimetric Activity Assay Kit (Sigma-Aldrich), following the manufacturer’s instructions. The activity was measured in 5-fold diluted sera for a period of 60 min and calculated against the provided standards.

### Liver SDH_2_ activity quantification

The Succinate Dehydrogenase (SDH_2_) Activity Colorimetric Assay Kit (MAK197, Sigma-Aldrich) was used to measure the hepatic succinate dehydrogenase activity. We manually homogenised 5 mg of frozen liver in 50 μl ice-cold SDH assay buffer (Sigma-Aldrich). Measurements (absorbance 600 nm) were made every 5 min for 40 min.

### Isolation and culture of primary hepatocytes and non-parenchymal cells

Primary hepatocytes from WT and MCJ-KO mice were isolated by collagenase perfusion of the liver as described previously.^25^ Hepatocytes and non-parenchymal cells were isolated from healthy and bile-duct ligated WT mice according to the protocol described in Zubiete-Franco *et al.*^28^ The cell viability was validated using the Trypan blue exclusion test, and greater than 80% viability was considered for the experiments.

### Protein isolation and Western blotting

The extraction of the total protein from liver tissue and hepatocytes was as described previously.^29^ The mouse anti-MCJ monoclonal antibody was generated as previously described.^20^^,^^30^ A description of the antibodies used is provided in Table S1. As a loading control, we used glyceraldehyde-3-phosphate dehydrogenase (Abcam), tubulin (Sigma-Aldrich), or β-actin (Sigma-Aldrich) antibody. As secondary antibodies, we used anti-rabbit-IgG-HRP-linked (Cell Signalling) and anti-mouse-IgG-HRP-linked (Santa Cruz Biotechnology) antibodies. Immunoreactive proteins were detected by Western Lightning Enhanced Chemiluminescence reagent (ECL, Perkin Elmer) or Clarity Western ECL Substrate (BioRad) and exposed to X-ray films (Amersham) in a Curix 60 Developer (AGFA). The bands were quantified by densitometry using the free image processing software ImageJ (http://rsbweb.nih.gov/ij).

### RNA isolation and quantitative real-time PCR

The RNA was isolated using Trizol reagent (Invitrogen), and the concentration and integrity were determined using Nanodrop. We treated 1–2 μg of total RNA with DNAse (Invitrogen) and reverse transcribed the RNA into cDNA using M-MLV Reverse Transcriptase (Invitrogen). RT-PCR was performed using SYBR® Select Master Mix (Applied Biosystems) and the Viia 7 Real-Time PCR System (Applied Biosystems). The Ct values were extrapolated to a standard curve, and the data were then normalised to the house-keeping gene (*Gapdh*). The sequences of the primers are described in Table S2.

### Cell transfection

WT hepatocytes were transfected with 2 μg of expression plasmid cDNA using the Jetprime reagent (Polyplus). The plasmids used for transfection were: pcDNA3-LacZ (Invitrogen) as negative controls, shMCJ, and pCMV6-MCJ. The transfection efficiency was confirmed by Western blotting.

### GCDCA and DCA treatment

WT and MCJ-KO hepatocytes were treated with toxic doses of glycochenodeoxycholic acid (GCDCA) or deoxycholic acid (DCA). We seeded 500,000 cells in 6-well plates, cultured overnight in 0% FBS MEM 1% PSG medium and treated the day after. The administered dose of GCDCA (Sigma) and DCA (Sigma) was 100 μM using ethanol as a vehicle.

### Apoptosis measurement

The caspase-3 activity was measured in the cells as previously described.^31^ The cells were lysed in caspase buffer and the protein content was determined using a Bradford protein assay. Twenty microlitres of 25× reaction buffer was mixed with 2.5 μl of fluorogenic caspase-3 substrate (Enzo Life Sciences, USA) and with 10–50 μg of protein lysate in a total volume of 500 μl. Readings were taken using a Spectramax M3 spectrophotometer (excitation wavelength 390 nm, emission wavelength 510 nm).

### Mitochondrial ROS determination

Mitochondrial ROS (mROS) production was measured with fluorescence microscopy using MitoSOX™ Red mitochondrial superoxide (Life Technologies), following the manufacturer’s instructions.

### Intracellular ATP levels

The ATP levels in the primary hepatocytes were determined using the ATPlite™ luminescence ATP detection assay system (Perkin Elmer) following the manufacturer’s recommendations. The final values were normalised to the total protein concentration.

### Statistical analysis

SPSS Statistics software v19.0 (IBM, Armonk, NY, USA) was used for the statistical analysis. The Kolmogorov–Smirnov test was used to check the normal distribution of the variables. For the statistical analysis of variables with a normal distribution, the Student *t* test or ANOVA was used, whereas for variables with a non-normal distribution, the Mann–Whitney *U* test was used. The categorical variables were compared using the Chi square test or Fisher’s exact test. A *p* value <0.05 was considered significant.

## Results

### Elevated hepatic MCJ levels in clinical and pre-clinical models of cholestasis

**Fig. 1:**
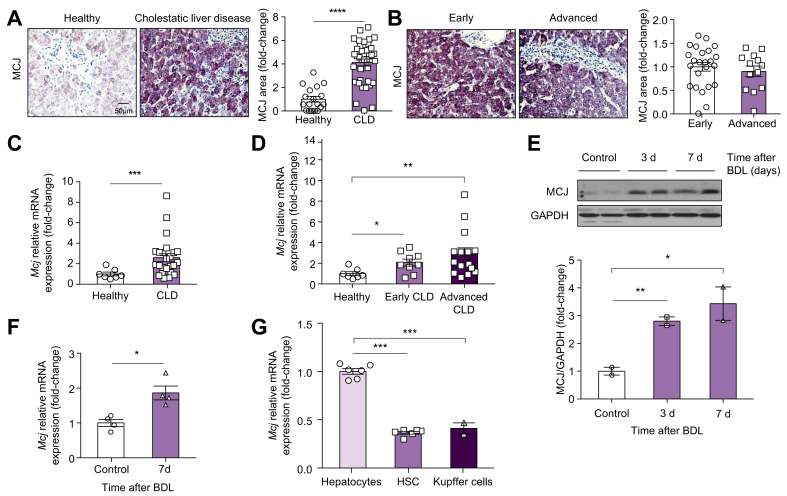
MCJ expression is increased in cholestasis. (A) Liver biopsies from individuals with histologically normal liver (healthy) (n = 18) and from patients with CLD (n = 37) where MCJ expression was determined by immunohistochemistry and quantified. (B) The same liver biopsies where MCJ expression by immunohistochemistry was analysed according to disease stage. (C) MCJ mRNA expression in liver tissue from other cohort subjects (healthy, n = 7; and patients with CLD, n=25). (D) MCJ mRNA expression in liver tissue from patients with CLD according to disease stage. (E) MCJ levels by Western blotting in WT liver extracts. (F) MCJ mRNA expression was determined by qPCR in livers from WT mice. (G) MCJ mRNA expression of hepatocytes and non-parenchymal cells (Kupffer cells and hepatic stellate cells -HSC-). Values are represented as mean ± SEM. ∗*p* <0.05; ∗∗*p* <0.01; ∗∗∗∗*p* <0.0001 (Student *t* test). BDL, bile duct ligation; CLD, cholestatic liver disease; GAPDH, glyceraldehyde-3-phosphate dehydrogenase; HSC, hepatic stellate cells; MCJ, methylation-controlled J; WT, wild-type.

The mitochondrial negative regulator MCJ was previously reported to be overexpressed in patients diagnosed with drug-induced liver injury.^22^ To study whether it could also be involved in CLD, the MCJ expression was assessed in a cohort of human samples of both healthy liver donors and patients with chronic CLD (PBC and PSC). The characteristics of the patients are shown in Table 1. A detailed study of the MCJ levels revealed that MCJ was significantly overexpressed in the livers derived from patients with CLD compared with healthy livers, at the protein (Fig. 1A) and mRNA levels (Fig. 1C). The MCJ levels were also evaluated at different disease stages, but no differences were found between the early (stage I and II of Ludwig and Scheuer) and the advanced disease (stage III and IV of Ludwig and Scheuer) (Fig. 1B and D).

Of the two main CLDs, PBC is best classified as an autoimmune disease because of its predominance in women, its association with other autoimmune diseases and its relationship with antimitochondrial M2 antibodies (AMA-M2). AMA-M2 are sensitive and specific for the diagnosis of PBC. These AMAs are directed against a family of mitochondrial enzymes, the E2 component of the pyruvate dehydrogenase complexes, which leads to the attack of biliary epithelial cells. Therefore, the evidence supports the antimitochondrial response as a direct effector of the liver pathology.^32^

For this reason, given the autoimmune character of PBC, the mitochondrial location of MCJ and its repressive effect on the respiratory chain, we assessed the presence of anti-MCJ Abs in serum samples from 205 patients with CLD using ELISA. The characteristics of these patients are shown in Table 2. Anti-MCJ appeared only in patients with CLD in a considerable percentage, although no differences were found in the clinical data and biochemical markers between the patients positive or negative for MCJ antibodies (data not shown). Specifically, 35 (17.1%) patients showed positive reactivity for anti-MCJ, 27 with PBC, and 8 with PSC, whereas no reactivity was identified in healthy individuals.

To determine the role of MCJ in cholestasis-induced liver injury, we performed BDL in 3-month-old WT and MCJ-KO mice. The MCJ expression at the protein level increased over time after BDL (Fig. 1E). At 7 days after BDL, the hepatic mRNA levels of MCJ were also regulated (Fig. 1F) and interestingly, when the parenchymal and non-parenchymal cells were isolated, MCJ was mainly expressed at the mRNA level (Fig. 1G) in hepatocytes.

Overall, we provide evidence that hepatic MCJ expression was increased during CLD, suggesting that MCJ could play a pathogenetic role in CLD.

### MCJ deficiency attenuated the inflammatory injury induced by BDL in mice

After BDL, bile acids accumulated rapidly in the liver producing significant hepatocellular necrosis and inflammation, particularly by infiltration of macrophages and neutrophils.^5^ Previous studies have shown that BA concentrations increased in the liver (27-fold) and serum (1,400-fold) within 6 h of surgery and remained elevated for up to 14 days.^33^ Under basal conditions, no significant differences in the liver content of BAs in WT and MCJ-KO mice were found. BDL induced a marked elevation in the total BAs that was similar in both groups of mice at 48 h after BDL (Fig. S1A). As there are BAs that are considered more toxic than others, we screened the different species of hepatic BAs and found no significant differences between the WT and MCJ-KO in serum and liver, at basal and 48 hours after BDL (Fig. S1B–D).

**Fig. 2:**
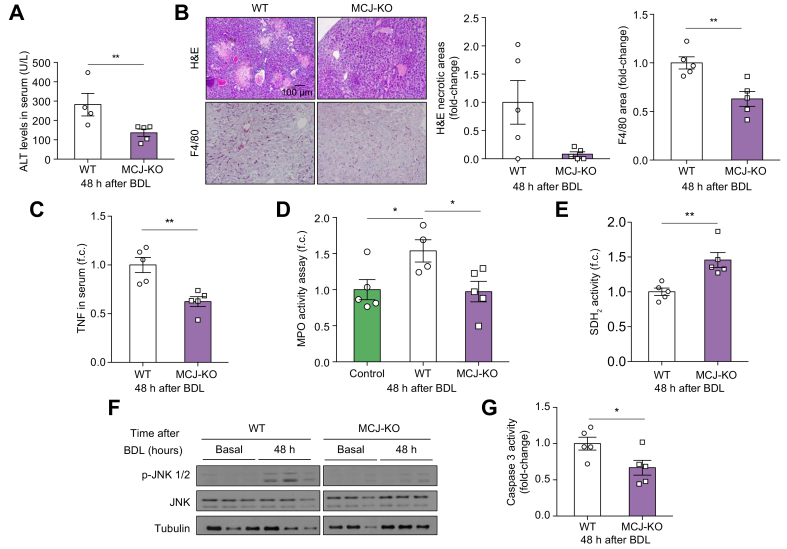
MCJ deficiency attenuates acute cholestasis-induced liver injury *in vivo*. WT (n = 5) and MCJ-KO (n = 5) mice were subjected to BDL. Liver injury was evaluated 48 hours after the procedure. (A) Serum ALT levels in WT and MCJ-KO 48 h after BDL. (B) Biliary infarcts were assessed by H&E staining, liver inflammation by F4/80 staining, and fibrogenesis by α-SMA staining. (C) Serum TNF levels in WT and MCJ-KO 48 h after BDL. (D) Systemic neutrophil activation evaluated by MPO activity. (E) Assessment of complex II activity by colourimetry with an SDH_2_ activity assay kit. (F) JNK activation by Western blotting in liver extracts. (G) Apoptosis measured by caspase-3 activity. Values are represented as mean ± SEM. ∗*p* <0.05; ∗∗*p* <0.01 (Student *t* test). α-SMA, alpha-smooth muscle actin; BDL, bile duct ligation; CLD, cholestatic liver disease; JNK, c-Jun N-terminal kinase; KO, knockout; MCJ, methylation-controlled J; MPO, myeloperoxidase; SDH_2_, succinate dehydrogenase; TNF, tumour necrosis factor; WT, wild-type.

To investigate the course of hepatocellular injury following BDL, the serum ALT levels were determined, and a histological quantification of biliary infarcts was performed. In spite of a similar hepatic BA profile 48 h after BDL, when compared with WT mice, there was a lower increase in ALT levels, and fewer biliary infarcts in MCJ-KO mice (Fig. 2A and B). Concomitantly, we found a reduction of the hepatic inflammatory infiltrate in MCJ-KO mice compared with WT mice as determined by the presence of F4/80 positive cells (Fig. 2B).

A decreased systemic inflammation in these MCJ-KO mice was also observed, supported by lower serum levels of TNF (Fig. 2C). MCJ deficiency resulted in reduced MPO activity in the peripheral blood compared with the baseline levels in comparison to WT mice under the BDL procedure (Fig. 2D). No changes were detected in the neutrophil numbers as analysed by flow cytometry in MCJ-KO mice *vs.* WT (Fig. S2A and B).

There is considerable evidence that neutrophil-derived ROS are the primary insult that leads to secondary mitochondrial dysfunction ending in the activation of stress kinases and cell death pathways (apoptosis and necroptosis).^13^^,^^34^ To assess the mitochondrial functionality at 48 h after BDL, we measured the activity of a component of the mitochondrial respiratory chain complex II, succinate dehydrogenase (SDH_2_). We found decreased SDH_2_ activity in WT mice compared with MCJ-KO mice (Fig. 2E).

In agreement, MCJ-KO mice showed a lower expression of the *Ucp2, Hif1a*, and *Ezh2* genes, which are directly related to ROS-induced liver damage and the reduction of mitochondrial biogenesis (*Pgc1a, Pgc1b, Tfam*, and *Nrf1*) (Fig. S2C), suggesting reduced ROS-induced mitochondrial damage. Minimal phosphorylation of c-Jun N-terminal kinase (JNK) was also detected in MCJ-KO liver extracts at the same time point (Fig. 2F). A lower detection of apoptosis in MCJ-KO liver tissue was demonstrated using a caspase-3 activity assay (Fig. 2G). No changes in the necroptotic response were detected at this time point in the absence of MCJ (data not shown).

### MCJ deficiency prevented the programmed cell death induced by BDL in mice

**Fig. 3:**
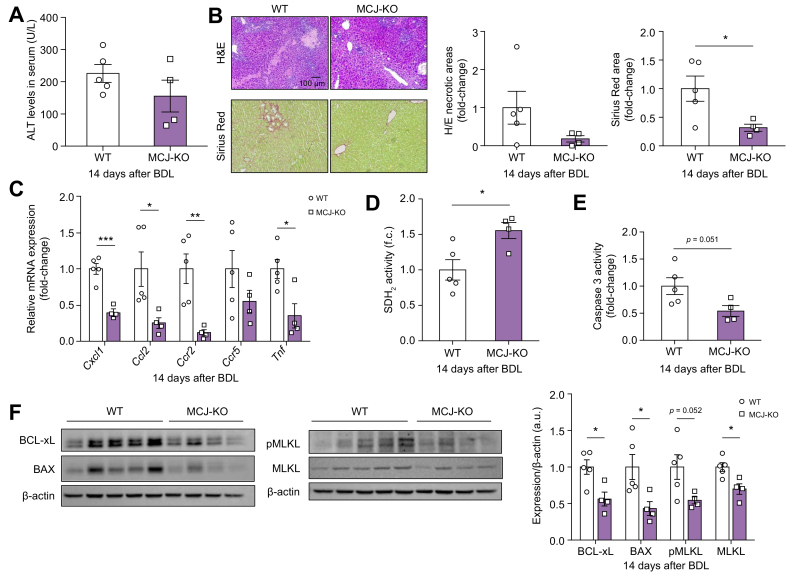
MCJ deficiency prevents chronic cholestasis-induced liver injury *in vivo*. WT (n = 5) and MCJ-KO (n = 4) mice were subjected to BDL. Liver injury was evaluated 14 days after the procedure. (A) Serum ALT levels in WT and MCJ-KO 14 days after BDL. (B) Biliary infarcts were assessed by H&E staining, and fibrogenesis by α-SMA staining 14 days after BDL. (C) Relative mRNA expression of inflammatory related genes in liver tissue. (D) Assessment of complex II activity by colourimetry with SDH_2_ activity assay kit. (E) Apoptosis measured by caspase-3 activity. (F) Expression of proteins related to apoptosis and necroptosis by Western blotting in WT and MCJ-KO liver extracts at 14 days after BDL. Values are represented as mean ± SEM. ∗*p* <0.05; ∗∗*p* <0.01; ∗∗∗*p* <0.001 (Student *t* test). α-SMA, alpha-smooth muscle actin; ALT, alanine aminotransferase; BAX, BCL2 associated X; BDL, bile duct ligation; KO, knockout; MCJ, methylation-controlled J; MLKL, mixed-lineage kinase domain-like pseudokinase; p-MLKL; phosphor-MLKL; SDH_2_, succinate dehydrogenase; WT, wild-type.

To evaluate the difference in liver injury during chronic cholestasis, we examined the liver 14 days after BDL. The ALT levels and biliary infarcts in MCJ-KO mice 14 days after BDL were still lower than in WT mice (Fig. 3A and B). At this time point of BDL, we found gene regulation of the hepatic expression of inflammatory cytokines (Tnf, Cxcl1, and Ccl2) and chemokine receptors (Ccr2 and Ccr5) (Fig. 3C). We also detected decreased SDH_2_ activity and increased apoptosis in WT mice compared with the MCJ-KO mice (Fig. 3D and E). Decreased apoptosis pathway activation in the liver of MCJ-KO mice 14 days after BDL was further supported by lower levels of Bcl-2 family proteins (BAX and BCL-xL) (Fig. 3F). In addition, necroptosis, a death process that releases inflammatory mediators, was significantly reduced in MCJ-KO mice compared with WT mice as determined by the protein levels of MLKL (Fig. 3C).

Previous reports showed that the liver fibrosis peak in the BDL model appeared at 14 days;^6^^,^^9^ therefore, we performed an immunohistochemical analysis for Sirius Red, a marker for collagen fibres. Staining levels were reduced in MCJ-KO mice compared with WT (Fig. 3B).

Overall, these data indicate that MCJ silencing is hepatoprotective, reduces cell death, affects mitochondria activity, and prevents the generation of liver inflammation and fibrosis after BDL.

### MCJ deficiency and *in vitro* silencing attenuated BA-induced hepatocyte injury

**Fig. 4:**
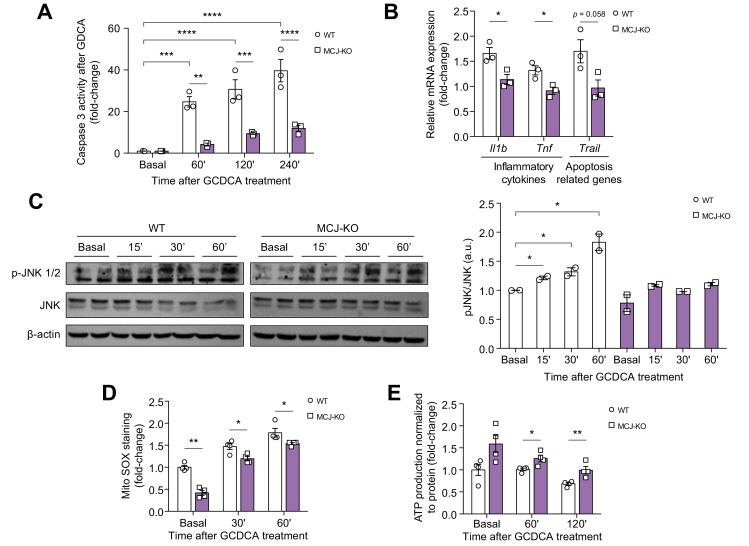
Lack of MCJ protects against bile acid-mediated toxicity in hepatocytes. WT and MCJ-KO hepatocytes were treated with GCDCA 100 μM. Duplicates, triplicates, or quadruplicates were used for each experimental condition. (A) Apoptosis measured by caspase-3 activity. (B) Relative mRNA expression in hepatocytes of different inflammatory and apoptosis-related genes after 1-h GCDCA treatment. (C) JNK activation by Western blotting. (D) mROS in primary WT and MCJ-KO hepatocytes determined by staining with MitoSOX reagent. (E) Total ATP levels in primary WT and MCJ-KO hepatocytes. Values are represented as mean ± SEM. ∗*p* <0.05; ∗∗*p* <0.01; ∗∗∗*p* <0.001; ∗∗∗∗*p* <0.0001 (Student *t* test). GCDCA, glycochenodeoxycholic acid; JNK, c-Jun N-terminal kinase; KO, knockout; MCJ, methylation-controlled J; ROS, reactive oxygen species; WT, wild-type.

As MCJ is mainly expressed in hepatocytes, in our preclinical model, we next evaluated the effect of MCJ in isolated mouse hepatocytes. Isolated primary hepatocytes from WT and MCJ-KO mice were treated with GCDCA 100 μM, a hydrophobic BA. One hour after GCDCA treatment, marked toxicity features were observed in WT hepatocytes, while MCJ-KO hepatocytes were significantly protected, as assessed by caspase-3 activity (Fig. 4A). The protection of MCJ-KO mice was further supported by the lower mRNA levels of inflammatory cytokines (*Il1b* and *Tnf*) and apoptosis inducer genes (tumour necrosis factor ligand superfamily member 10 [*Trail*]) (Fig. 4B).

As observed in the hepatic tissue, JNK activation directly correlated with cell death. We detected a lower phosphorylation of JNK in MCJ-KO hepatocytes when compared with WT hepatocytes after GCDCA treatment (Fig. 4C). A direct effect on the mitochondrial membrane was attributed to BAs, and could be incorporated even in open pores in the membrane disturbing the mitochondrial permeability and membrane potential,^10^^,^^17^ affecting the mitochondrial function.

The mitochondrial permeability transition (MPT), a crucial factor in cholestatic liver injury, is directly related to ROS.^35^^,^^36^ Reduced levels of mitochondrial ROS were detected in hepatocytes lacking MCJ after GCDCA treatment (Fig. 4D). Finally, 1 of the main functions of mitochondria is to provide energy (ATP) through oxidative phosphorylation. ATP production is used as an indicator of mitochondrial functionality. Importantly, MCJ-KO hepatocytes had lower ATP depletion compared with WT hepatocytes as shown in Fig. 4E.

Additionally, we studied the primary hepatocyte response to treatment with another BA, DCA. DCA is a hydrophilic BA that decreases the activity of several enzyme complexes of the electron transport chain.^10^ We observed the results superimposable on those shown with GCDCA (Fig. S3A–E). MCJ was transiently repressed in WT hepatocytes with shRNA (shMCJ). Under these circumstances, MCJ silencing reduced the DCA-induced cell death (Fig. S3F). On the contrary, MCJ overexpression in MCJ-KO hepatocytes resulted in increased susceptibility to DCA-induced injury as detected by increased caspase-3-activity (Fig. S3G).

Hence, after toxic BA administration, the absence of MCJ maintained the hepatocyte mitochondrial function, reducing ROS production, and attenuated against the fall in ATP levels.

### *In vivo* silencing of MCJ ameliorated cholestasis-induced liver injury

**Fig 5:**
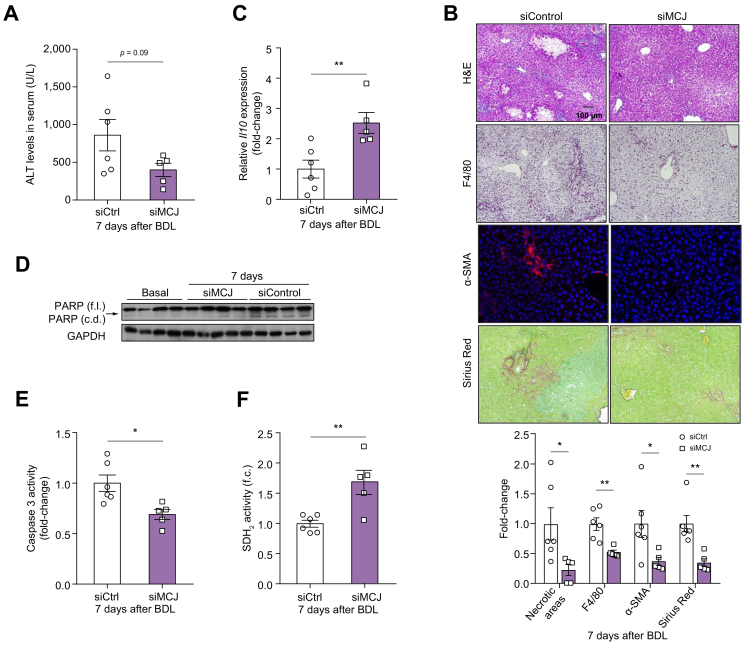
MCJ *in vivo* silencing counteracts cholestasis-induced liver injury. BDL was performed on WT mice, and 3 and 5 days later, siControl (n = 6) or si*Mcj* (n = 5) was injected i.v. into these mice. (A) Serum ALT levels in siControl and si*Mcj*-treated animals. (B) Biliary infarcts were assessed by H&E staining, inflammation by F4/80 staining, fibrogenesis by α-SMA staining, and connective tissue by Sirius Red staining. (C) Relative mRNA expression of different inflammatory genes. (D) PARP (full length [f.l.] and catalytic domain [c.d.]) by Western blotting. (E) Apoptosis measured by caspase-3 activity. (F) Assessment of complex II activity by colourimetry with an SDH_2_ activity assay kit. Values are represented as mean ± SEM. ∗*p* <0.05; ∗∗*p* <0.01 (Student *t* test). α-SMA, alpha-smooth muscle actin; ALT, alanine aminotransferase; BDL, bile duct ligation; GAPDH, glyceraldehyde-3-phosphate dehydrogenase; KO, knockout; MCJ, methylation-controlled J; PARP, poly (ADP-ribose) polymerase; SDH_2_, succinate dehydrogenase; WT, wild-type.

Recent clinical trials employing siRNA approaches demonstrated an efficient and durable downregulation of the target gene in the liver with promising clinical outcomes and little toxicity.^37^ To evaluate the therapeutic effectiveness of MCJ knockdown in CLD, we silenced MCJ using a specific siRNA against MCJ and used a scrambled siRNA as a control. Silencing was performed through i.v. injection at 3 and 5 days after BDL. The mice were sacrificed 7 days after BDL, and liver injury was assessed. The efficiency of MCJ silencing was evaluated using Western blotting (Fig. S4A). The ALT levels in the serum tended to decrease in mice treated with si*Mcj* and minimal biliary infarcts were detected compared with the control mice (Fig. 5A and B).

Evaluation of the inflammatory response using F4/80 immunostaining showed a lower macrophage infiltration in mice administered with si*Mcj* (Fig. 5B). The observed reduction of the hepatic inflammatory response in the absence of MCJ was further supported by the upregulation of the anti-inflammatory cytokine *Il10* (Fig. 5C). Likewise, although siControl mice showed increased cleavage of poly(ADP-ribose) polymerase (PARP), a death substrate for caspase-3 and a marker of cells undergoing apoptosis, this was not observed in si*Mcj*-treated mice (Fig. 5D and Fig. S4B). Significantly reduced caspase-3 activity was also found in mice lacking MCJ compared with WT mice (Fig. 5E). Besides, improved mitochondrial activity was observed in mice lacking MCJ as increased SDH_2_ activity was measured (Fig. 5F).

Liver fibrosis is a critical endpoint of murine models reflecting chronic cholestatic injury. Seven days after BDL, the expression of α-SMA, a marker of activated hepatic stellate cells, myofibroblasts and hydroxyproline, and the quantification of Sirius Red staining, which stains the connective tissue intense red, were significantly lower in mice treated with si*Mcj* (Fig. 5B).

In summary, the loss of MCJ not only protected against, but also ameliorated cholestasis-induced hepatic injury.

## Discussion

Excessive concentrations of certain hydrophobic BAs are known to induce injury to hepatocytes,^38^^,^^39^ further exacerbating hepatic inflammation and liver fibrosis.^40^ Currently, available therapies for cholestasis are scarce. Several studies suggested a major role of mitochondria in the pathophysiology of cholestatic liver injury.^18^^,^^19^ Indeed, previous studies demonstrated a pivotal role of proteins encoded by mitochondrial DNA in BA-mediated apoptosis activation.^41^

Mitochondria are the main source of energy in hepatocytes as they are responsible for ATP synthesis through oxidative phosphorylation by the ETC.^42^ This metabolic process of oxidative phosphorylation plays a key role in the onset and progression of liver diseases as it is an important source of ROS. This is partly counter-balanced by the concomitant accumulation of other cholephilic compounds, such as bilirubin and biliverdin, which play a protective role against BA-induced oxidative stress in liver cells.^43^ Here, we focused our study on MCJ, a mitochondrial protein encoded by nuclear DNA, which is the first endogenous inhibitor of the ETC.

Significantly higher levels of MCJ in the livers of patients with CLD and mice after BDL were found, which confirms the important role of MCJ in CLD pathogenesis. The increased levels of MCJ in cholestasis-mediated injury could function as an autoantigen to stimulate an autoimmune response, as already described for AMA in PBC. Here, we found that a non-negligible percentage of patients with CLD had serologic reactivity to MCJ protein. These antibodies did not appear to be associated with a disease stage, and their clinical significance remains to be determined through long-term follow-up.

Considering that MCJ levels are increased in patients with CLD as well as in preclinical mouse models of cholestasis, we evaluated the effect of MCJ depletion in experimental models. BDL is the most widely used preclinical model to induce cholestatic injury and liver fibrosis and an appropriate model to investigate the ongoing hepatic insults associated with cholestasis.^44^ Kupffer cells and systemic neutrophils have been shown to be activated during the early phases after BDL, and neutrophils negatively modulate the progression of liver injury during BDL.^5^

MPO is the most abundant proinflammatory enzyme stored in neutrophils and it is crucial to understand its effects in inflammation. Importantly, neutrophil activity measured by MPO in the absence of MCJ appeared to be downregulated in comparison with that in WT mice. Neutrophil-mediated ROS are the triggered response to mitochondrial dysfunction. Mitochondrial impairment leads to ROS overproduction and the release of cytochrome c activating the intrinsic apoptotic pathway.^45^ In this sense, we found higher complex II activity and lower Bcl-2 family protein levels (BAX and BCL-xL) in MCJ-KO mice compared with the WT after BDL.

Higher serum levels of TNF in WT mice also translate into a greater inflammatory response. TNF can lead to the further activation of cell death pathways, including necroptosis and extrinsic apoptosis.^46^ Afonso *et al.*^9^ detected high levels of MLKL, the effector protein in necroptosis, in the liver of patients with PBC, suggesting that necroptosis likely has an active role in disease triggering and progression. Here, we found that MCJ deficiency prevented both necroptosis and apoptosis after BDL.

Less apoptosis was also found in hepatocytes from MCJ-KO animals and MCJ silencing after treatment with high concentrations of toxic BAs. Hence, given the regulatory effect of MCJ on mitochondrial activity, we studied whether the protective action of MCJ silencing and depletion against BA-induced hepatocyte injury could be mediated by mitochondrial targeted effects. In this study, we observed an increased ROS production in WT hepatocytes *vs.* MCJ-KO hepatocytes after GCDCA treatment. Confirming this, we found less depletion in the ATP levels in MCJ-KO hepatocytes treated with GCDCA.

ETC complexes can interact to form respiratory supercomplexes or respirasomes. These formations bring together the complexes I, III, and IV, facilitating the transport of electrons and minimising their leakage and, hence, ROS production.^45^ MCJ has been shown to interfere with supercomplex formation in the heart, T cells, and the liver.[20], [21], [22] In our study, the toxic administration of BAs in hepatocytes lacking MCJ generated less ROS production than in WT hepatocytes; thus, we speculate that that toxic BAs promote the interaction of MCJ with complex I interfering in its association with complex III.

Additionally, ROS induce the expression of pro-inflammatory cytokines and activate mitogen-activated protein kinases (MAPKs) such as JNK.^34^ Accordingly, we found a lower expression of several inflammatory cytokines and a lower activation of JNK both in MCJ-KO hepatocytes under BA treatment and in MCJ-KO mice after BDL. Sustained activity of JNK contributes to the development of inflammation, fibrosis, and even carcinogenesis, and, in the liver, it is the dominant MAPK effector that catalyses the phosphorylation of numerous proteins.

p-JNK translocates into the mitochondria where it binds to proteins on the mitochondrial outer membrane causing MPT with consequent ATP depletion, release of proapoptotic factors, and hepatocyte death.^13^ In fact, inhibiting interactions between p-JNK and mitochondria and reducing mitochondrial ROS production reduced liver injury in mouse models.^34^ Thus, MCJ inhibition can reduce cholestasis-induced liver injury by blocking the ROS overproduction and JNK activation.

Focusing on a possible therapeutic approach, we showed that MCJ silencing protected from cholestasis-mediated injury in WT mice with BDL, which further highlights the role of MCJ in the pathogenesis of cholestasis. A lower expression of the MCJ protein was achieved with siRNA treatment aimed to suppress the expression of the MCJ gene, and the administration of this si*Mcj* was carried out when the inflammatory and fibrotic response was already initiated.

In summary, MCJ deficiency protected against the mitochondrial dysfunction caused by cholestasis, which led to reduced hepatic inflammation, oxidative stress, and cell death. Given our results, this study demonstrated that MCJ is an important regulator of the liver injury associated with the mitochondrial dysfunction that occurs in CLDs. Consequently, MCJ may be a therapeutic target to attenuate BA-induced liver injury and delay the progression of chronic cholestatic disease in those cases where the currently available therapies have proven insufficient.

## Financial support

This work was supported by grants from 10.13039/100000002NIH (10.13039/100000016US Department of Health and Human Services-R01AT001576 (SCL and MLM); ELKARTEK 2016, Departamento de Industria del Gobierno Vasco (to MLM); 10.13039/100014440Ministerio de Ciencia, Innovación y Universidades MICINN: SAF2017-87301-R, RTI2018-096759-1-100 and RTI2018-096494-B-100 integrado en el Plan Estatal de Investigación Científica y Técnica y Innovación, cofinanciado con Fondos 10.13039/501100002924FEDER (to MLM, TCD, and JA, respectively); 10.13039/501100002704Asociación Española contra el Cáncer (TCD, PF-T, and MLM-C); Daniel Alagille award from 10.13039/501100009253EASL (to TCD); 10.13039/501100002704Fundación Científica de la Asociación Española Contra el Cáncer (AECC Scientific Foundation) Rare Tumor Calls 2017 (to MLM); LCF/PR/HP17/52190004 10.13039/100010434La Caixa Foundation Program (to MLM); Umbrella Ayudas Fundación BBVA a Equipos de Investigación Científica 2018 (to MLM), Programa retos RTC2019-007125-1 (to MLM-C and JS), Proyectos Investigacion en Salud DTS20/00138 (to MLM-C and JS), and 10.13039/501100004587ISCIII (Immunomediated Nonalcoholic SteaTohepatItis; prevalence and CharacTerization. INSTInCT study -PI18/01304-) (to JC).

## Authors’ contributions

Conceptualisation: PI, NG-U, LB-T, JA, ML-H, TCD, MR, JC, MLM-C. Data curation: PI, NG-U, MS-M. Formal analysis: PI, NG-U. Investigation: PI, NG-U, LB-T, MS-M, DF-R, PF-T, VGdJ, SL-O, JS, MB, FL-O, MV-R, NE, MJM, JJGM, ML-H. Methodology: PI, NG-U, LB-T, TCD, MR, JC, MLM-C. Validation: PI, NG-U, MLM-C. Visualisation: PI, NG-U, LB-T. Writing – original draft: PI, NG-U, JC, MLM-C. Writing – review and editing: LB-T, MS-M, DF-R, MV-R, NE, JLC, SCL, MM, PM, JA, MJM, JJGM, ML-H, TCD, MR. Resources: MR, CF, JLC, SCL, MM, PM, JA, MR, JC. Project administration: JC, MLM-C. Supervision: JC, MLM-C. Funding acquisition: JC, MLM-C

## Data availability

The datasets generated during and/or analysed during the current study are included in this published article (and its Supplementary data) or available from the corresponding authors on reasonable request.

## Conflict of interest

Dr. María Luz Martínez-Chantar advises for Mitotherapeutix LLC. Dr. Javier Crespo reports grants and research support from Gilead Sciences, AbbVie, MSD, and Intercept Pharmaceuticals (all outside the scope of the submitted work). He is a speaker for Gilead Sciences and AbbVie.

Please refer to the accompanying ICMJE disclosure forms for further details.
