# Supplementary material for: Boosting mitochondria activity by silencing MCJ overcomes cholestasis-induced liver injury
Source: JHEP Rep. 2021 Mar 18;3(3):100276. doi: 10.1016/j.jhepr.2021.100276 (PMC8099785; doi:10.1016/j.jhepr.2021.100276)
Supplement: Supplementary file 1 [file mmc1.pdf]

Boosting mitochondrial activity by silencing MCJ overcomes cholestasis-induced liver injury

Paula Iruzubieta, Naroa Goikoetxea-Usandizaga, Lucía Barbier-Torres, Marina Serrano-Maciá, David Fernández-Ramos, Pablo Fernández-Tussy, Virginia Gutiérrez-de-Juan, Sofia Lachiondo-Ortega, Jorge Simon, Miren Bravo, Fernando Lopitz-Otsoa, Mercedes Robles, Carlos Ferre-Aracil, Marta Varela-Rey, Natalia Elguezabal, José Luis Calleja, Shelly C Lu, Malgorzata Milkiewicz, Piotr Milkiewicz, Juan Anguita, María J Monte, José J G Marin, Marcos López-Hoyos, Teresa C. Delgado, Mercedes Rincón, Javier Crespo, María Luz Martínez-Chantar

Table of contents

Supplementary methods 2
Supplementary material 3
Table S1 3
Table S2 4
Supplementary results 7
Fig. S1 7
Fig. S2 8
Fig. S3 9
Fig. S4 10

SUPPLEMENTARY METHODS

Hematoxylin & Eosin. Paraffin-embedded sections (5 µm thick) of formalin-fixed liver samples were deparaffinized in Histo-Clear and rehydrated through graded alcohol solutions. Once hydrated, the sections were placed in hematoxylin solution for 5 minutes and washed in running tap water for 5 minutes. Then, sections were placed in eosin solution for 15 minutes, washed in running tap water for 3 minutes, dehydrated, cleared, and mounted using DPX mounting medium.

Sirius red. Paraffin-embedded sections (5 µm thick) of formalin-fixed liver samples were deparaffinized in xylene and rehydrated through graded alcohol solutions. Once hydrated, the sections were placed in 0.01% Fast Green FCF picric acid solution for 15 minutes, in 0.04% Fast Green FCF/0.1% Sirius red picric acid solution for 15 minutes, dehydrated, and mounted using DPX mounting medium.

Immunohistochemistry. The sections were unmasked according to the primary antibody to be used and subjected to a peroxide block (3% H_2O_2 in 1x PBS) for 10 minutes at room temperature (RT). For staining with mouse-derived primary antibodies in mouse tissues, the samples were blocked with goat anti-mouse Fab fragment (Jackson Immunoresearch) (1 hour, RT, 1:10) before being blocked with serum (5% goat serum in 1X PBS) for 30 minutes at RT. Then, sections were incubated in a humid chamber with primary antibody in DAKO antibody diluent (DAKO) followed by Envision anti-rabbit or anti-mouse (DAKO) or ImmPRESS anti-rat (Vector) HRP-conjugated secondary antibody incubation for 30 minutes at RT.

The unmasking and incubation conditions for each antibody are shown in Table S1. Colorimetric detection was performed with Vector VIP (purple) chromogen (Vector), and the sections were counterstained with hematoxylin. The samples were dehydrated through graded alcohol solutions and Histo-Clear and mounted using DPX mounting medium. For αSMA staining, the sections were incubated with primary antibody conjugated to Cy3 and mounted with Fluoromount-G (Southern Biotech.) containing 0.7 mg/L of DAPI.

SUPPLEMENTARY MATERIAL

Table S1.

Table S1. Incubation conditions, dilution, supplier and catalog number for each antibody employed in this study for Western blotting.

	Incubation solution	Dilution	Supplier	Catalog Nº
**Anti-mouse IgG, HRP-linked**	TBS-Tween (0.1%)-milk (5%)	1:5000	Cell Signaling	7076
**Anti-rabbit IgG, HRP-linked**	TBS-Tween (0.1%)-milk (5%)	1:5000	Cell Signaling	7074
**β-actina**	TBS-Tween (0.1%)-milk (5%)	1:5000	Sigma-Aldrich	A5441
**BAX**	TBS-Tween (0.1%)-milk (5%)	1:1000	Cell Signaling	2772S
**BCL-xL**	TBS-Tween (0.1%)-milk (5%)	1:1000	Santa Cruz	sc-7195
**GAPDH**	TBS-Tween (0.1%)-milk (5%)	1:5000	Abcam	ab8245
**JNK**	TBS-Tween (0.1%)-milk (5%)	1:1000	Cell Signaling	9252S
**MCJ**	TBS-Tween (0.1%)-milk (5%)	1:500	Provided by Dra. Rincon	-
**MLKL**	TBS-Tween (0.1%)-milk (5%)	1:1000	MilliporeSigma	SAB1302339
**PARP**	TBS-Tween (0.1%)- milk (5%)	1:1000	Cell Signaling	9542

**pJNK (Thr183/Tyr185)**	TBS-Tween (0.1%)-milk (5%)	1:1000	Invitrogen	44682G
**pMLKL**	TBS-Tween (0.1%)-milk (5%)	1:1000	Abcam	ab196436
**Tubulina**	TBS-Tween (0.1%)-milk (5%)	1:5000	Sigma-Aldrich	T9026

GAPDH, Glyceraldehyde-3-phosphate dehydrogenase; HRP, Horseradish peroxidase; JNK, c-Jun N-terminal kinase; BAX, Bcl-2 associated X-protein; BCL-xL, B-cell lymphoma extra large protein; MCJ, Methylation-Controlled J; MLKL, Mixed lineage kinase domain pseudokinase; PARP, Poly (ADP-ribose) polymerase; p-JNK, phosphor-JNK; pMLKL, phosphor-MLKL; TBS, Tris-Buffered Saline.

Table S2.

Table S2. Sequence of primers used for RT-qPCR.

	Symbol	Species		Sequence
**9S ribosomal RNA**	9S	Mus musculus	Forward	5’-GACTCCGGAACAAACGTGAGG-3'
			Reverse	5’-CTTCATCTTGCCCTCGTCCA-3'
**C-X-C motif chemokine ligand 1**	Cxcl1	Mus musculus	Forward	5’- GGTGTCCCCAAGTAACGGAG-3'
			Reverse	5’-TTGTCAGAAGCCAGCGTTCA -3'
**C-C motif chemokine ligand 2**	Ccl2	Mus musculus	Forward	5’- GACCCCAAGAAGGAATGGGT -3'
			Reverse	5’- ACCTTAGGGCAGATGCAGTT -3'
**C-C motif chemokine receptor 2**	Ccr2	Mus musculus	Forward	5’- ATCCACGGCATACTATCAACAT -3'
			Reverse	5’- CAAGGCTCACCATCATCGTAG-3'

**C-C motif chemokine receptor 5**	Ccr5	Mus musculus	Forward<br>Reverse	5'- GTGTGGAAAATGAGGACTGCAT -3'<br>5'- GTCAGAACGGTCAACTTTGGG -3'
**Interleukin 10**	Il-10	Mus musculus	Forward<br>Reverse	5'-GGTTGCCAAGCCTTATCGGA-3'<br>5'-ACCTGCTCCACTGCCTTGCT-3'
**Methylation J-Controlled**	Mcj	Mus musculus	Forward<br>Reverse	5' -ACGCCGACATCGACCACACAG-3'<br>5'-AATCTTCCTTGCTGTTGCCGTC-3'
**Nuclear receptor factor 1**	Nrf1	Mus musculus	Forward<br>Reverse	5'-CTTCATGGAGGAGCACGGAG-3'<br>5'-CGTGGAGTTGAGGATGTCCC-3'
**Peroxisome proliferator-activated receptor gamma coactivator 1-alpha**	Pgc-1α	Mus musculus	Forward<br>Reverse	5'-AGACAGGTGCCTTCAGTTCAC-3'<br>5'-ACCAGAGCAGCACACTCTATG-3'
**Peroxisome proliferator-activated receptor gamma coactivator 1-beta**	Pgc-1β	Mus musculus	Forward<br>Reverse	5'-TCTGACGTGGACGAGCTTTC-3'<br>5'-CGTCCTTCAGAGCGTCAGAG-3'
**Transcription factor A, mitochondrial**	Tfam	Mus musculus	Forward<br>Reverse	5'-CTGCCTTCCTCTAGCCCGGG-3'<br>5'-GTAACAGCAGACAACTTGTG-3'
**Tumor necrosis factor**	Tnf	Mus musculus	Forward<br>Reverse	5'- CGTCAGCCGATTTGCTATCT-3'<br>5'-CGGACTCCGCAAAGTCTAAG -3'
**TNF-related apoptosis**	Trail	Mus musculus	Forward<br>Reverse	5'- CCAACGAGATGAAGCAGC-3'<br>3'- CCATCAGTGGAGTCCCAG-3'

**inducing ligand**				
**Uncoupling protein 2**	Ucp2	Mus musculus	Forward	5’-AAAGCAGCCTCCAGAACTCCG-3’
			Reverse	5’-TGGAGAAACGGGGACCTTCA-3’

SUPPLEMENTARY RESULTS

Fig. S1.

a

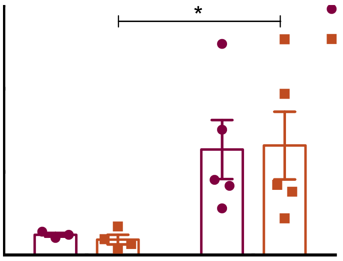

b

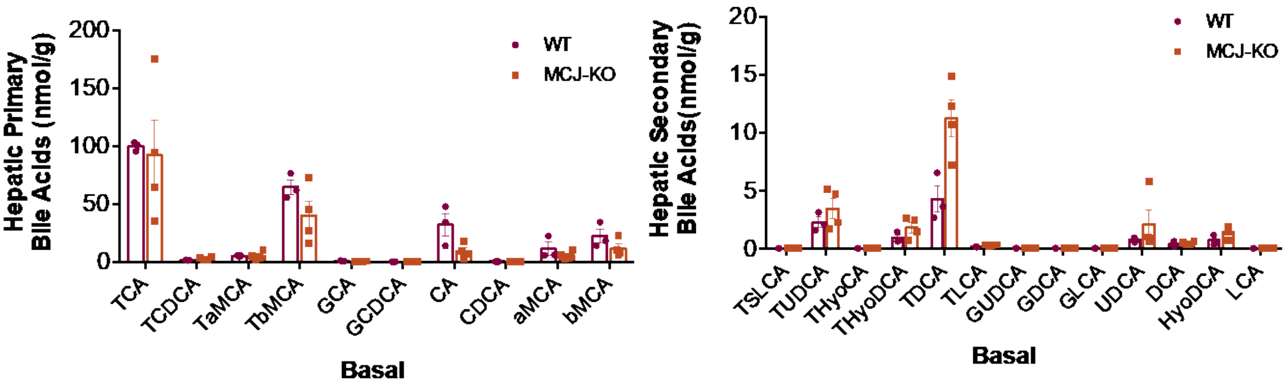

c

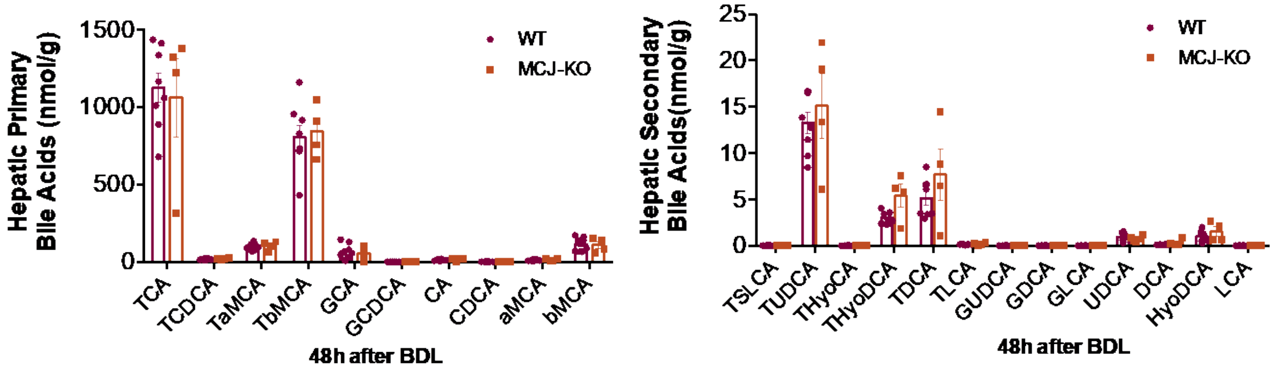

d

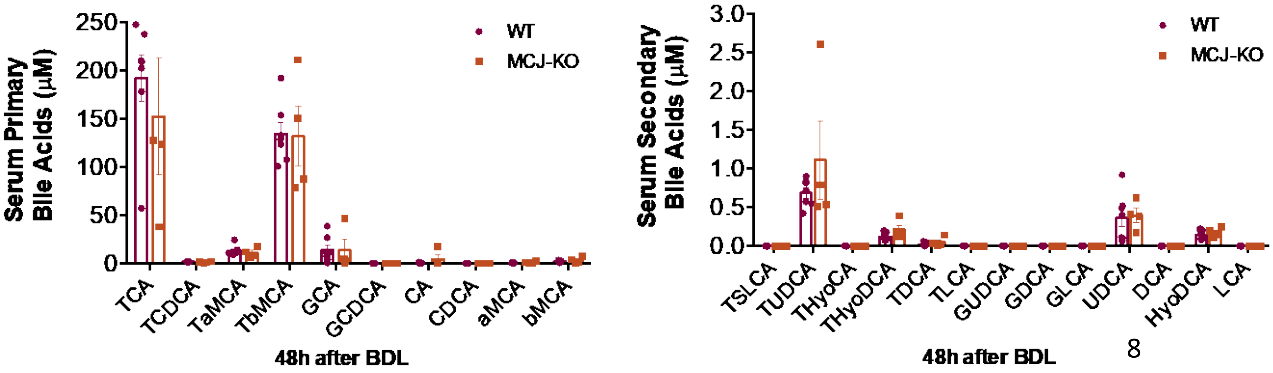

Fig. S1. Concentration of different species of bile acids in serum and in liver tissue from WT and MCJ-KO mice, in basal and after 48h of BDL. (a) Concentration of total bile acids in liver tissue from basal WT (n=3) and MCJ-KO (n=4) vs after 48 hours of BDL WT (n=8) and MCJ-KO (n=4). **(b)** Concentration of primary and secondary bile acids in liver tissue from basal WT (n=3) and MCJ-KO (n=4). **(c)** Concentration of primary and secondary bile acids in liver tissue from WT and MCJ-KO after 48h of BDL **(d)** Concentration of primary and secondary bile acids in serum from WT (n = 8) and MCJ-KO (n = 4) after 48h of BDL. Values are represented as mean ± SEM and Student's t-test was used to compare groups. *p<0.05 (Student's t test).

Fig. S2.

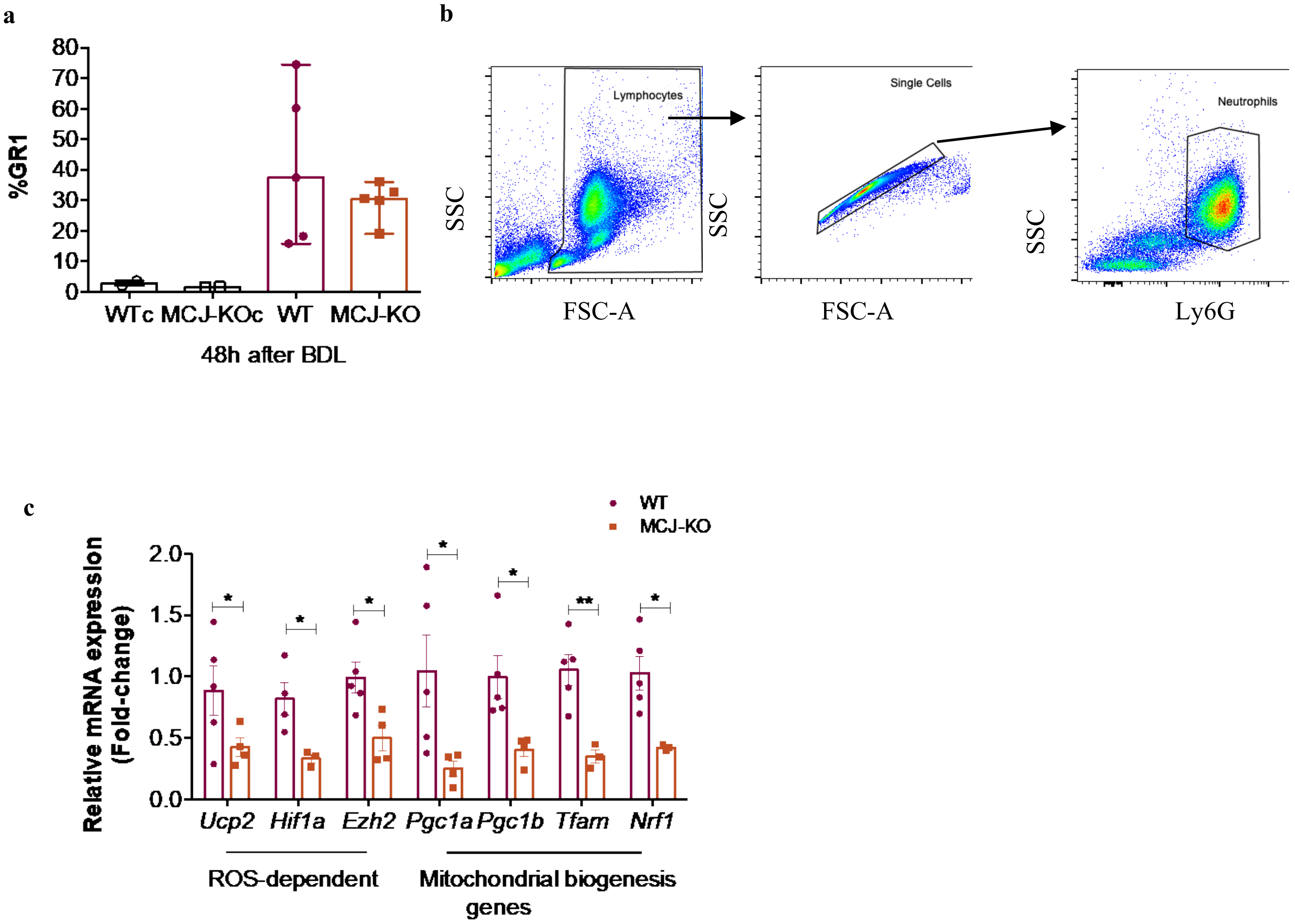

Fig. S2. MCJ deficiency prevents neutrophil activation and protects against mitochondrial dysfunction. WT (n = 5) and MCJ-KO (n = 5) mice were subjected to BDL. **(a)** Percentage of GR-1+ cells in WT and MCJ deficient mouse peripheral blood after 48 h of BDL, relative to the lymphocyte population. Age-matched WT and MCJ-deficient mice controls were also analyzed (2 mice each). **(b)** Gating strategy to identify the neutrophil population in murine peripheral blood. **(c)** Relative mRNA expression of different ROS-dependent and mitochondrial biogenesis-related genes in liver tissue from WT and MCJ-KO

mice, after 48 hours of BDL. Values are represented as mean ± SEM. *p<0.05; **p<0.01 (Student’s t test).

Fig. S3.

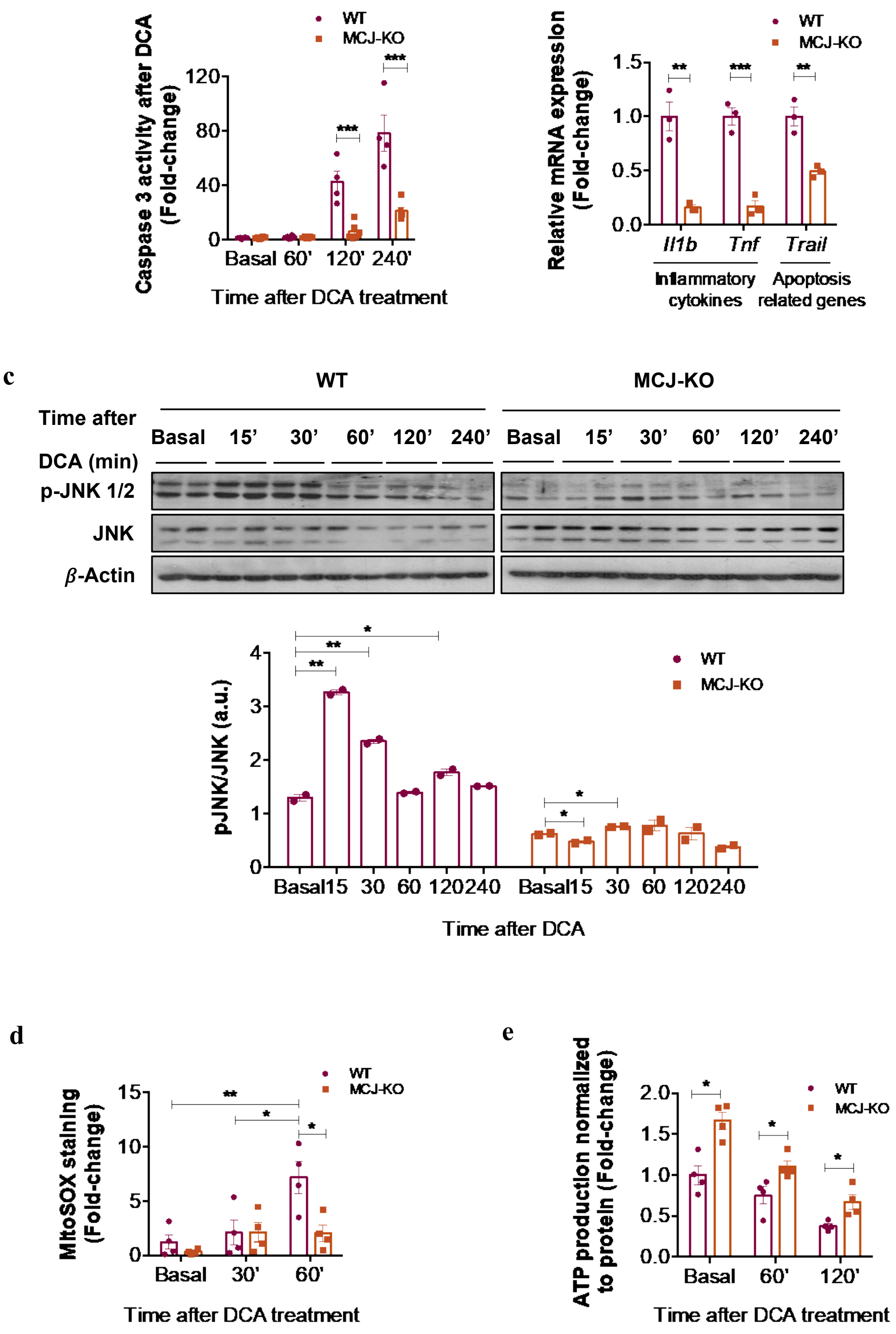

f

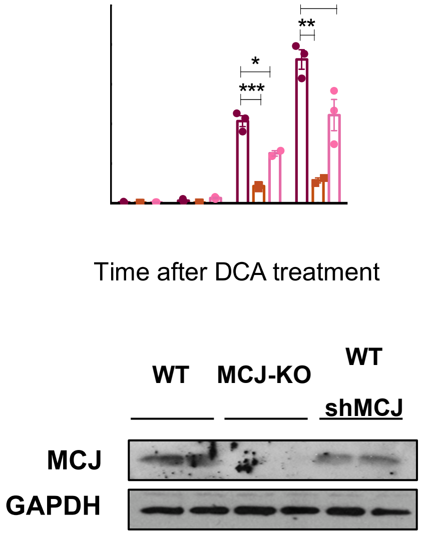

g

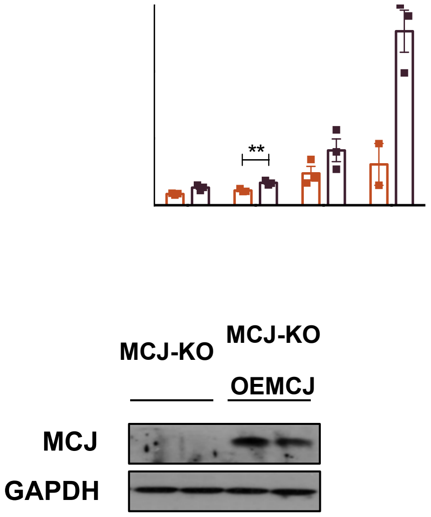

Fig. S3. Lack of MCJ protects against DCA-mediated toxicity in hepatocytes. WT and MCJ-KO hepatocytes were treated with DCA 100 µM. Quadruplicates or triplicates were used per experimental condition **(a)** Hepatocyte death measured by caspase-3 activity. **(b)** Relative mRNA expression in hepatocytes of different inflammatory and apoptosis related genes after 1-hour DCA treatment. **(c)** JNK activation by western blotting. **(d)** mROS in primary WT and MCJ-KO hepatocytes determined by staining with MitoSOX reagent. **(e)** Total ATP levels in primary WT and MCJ-KO hepatocytes. **(f)** Cell death in WT and ShMCJ hepatocytes. MCJ silencing was evaluated by western blotting. **(g)** Cell death in MCJ-KO and MCJ-KO OE hepatocytes. MCJ overexpression was evaluated by western blotting. Values are represented as mean ± SEM. *p<0.05; **p<0.01; ***p<0.001; ****P<0.0001 (Basal vs DCA and WT vs MCJ-KO) (WT vs MCJ-KO or WT shMCJ) (MCJ-KO vs MCJ-KO OEMCJ) (Student's test).

Fig. S4.

a

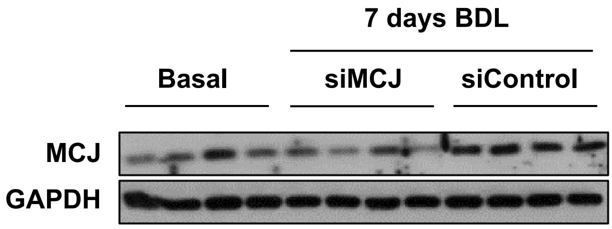

b

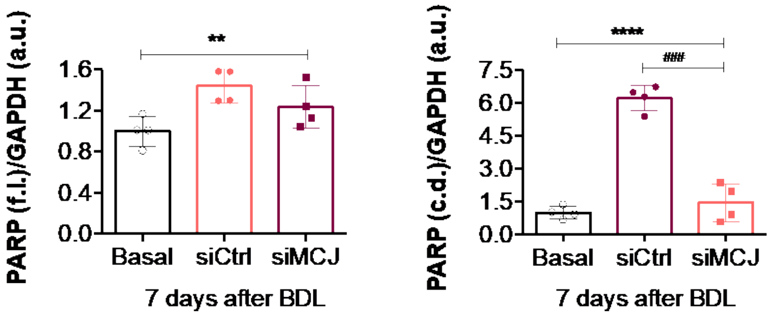

Fig. S4. WT mice basal and 7 days after BDL. WT mice with BDL were treated with MCJ-specific siRNA (siMCJ) or control (siControl). (a) MCJ silencing was evaluated by western blotting. **(b)** Densitometric analysis of PARP protein expression by western blotting. Values are represented as mean ± SEM. **p<0,01; ****p<0,0001 (Basal vs siControl); ###p<0,001 (siMCJ vs siControl) (Student's t test).

ORIGINAL WESTERN BLOTS

Figure 1E

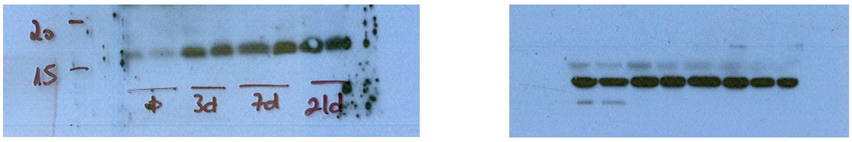

MCJ GAPDH

Figure 2F

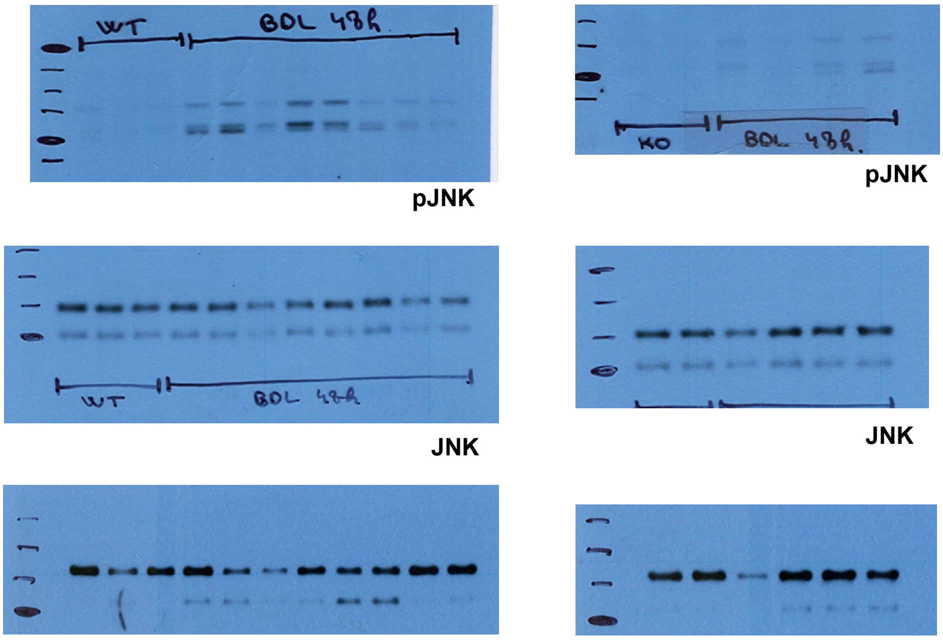

pJNK pJNK

JNK JNK

Tubulin Tubulin

Figure 3F

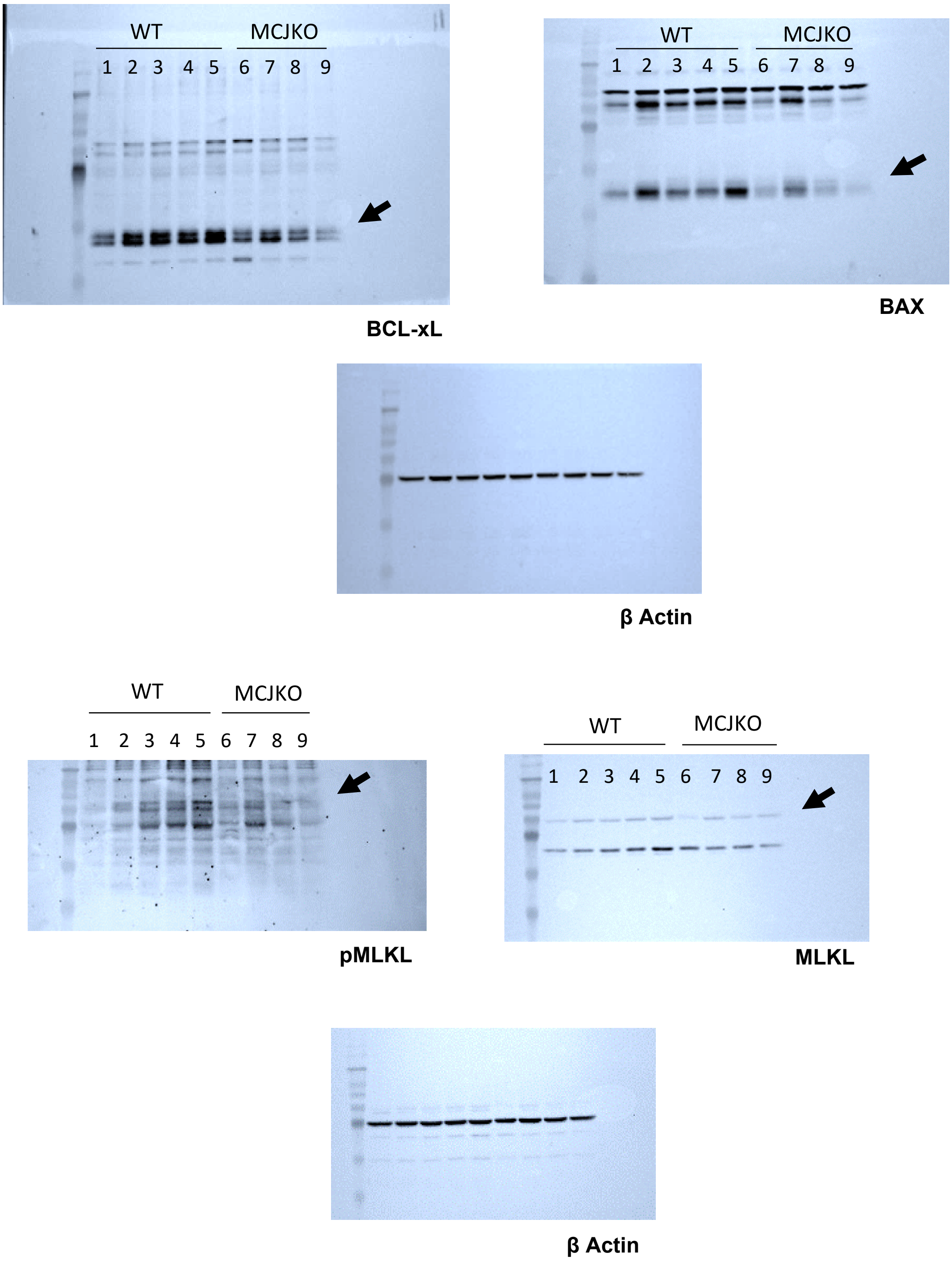
WT
MCJKO
1 2 3 4 5 6 7 8 9
BCL-xL
WT
MCJKO
1 2 3 4 5 6 7 8 9
BAX
β Actin
WT
MCJKO
1 2 3 4 5 6 7 8 9
pMLKL
WT
MCJKO
1 2 3 4 5 6 7 8 9
MLKL
β Actin

Figure 4C

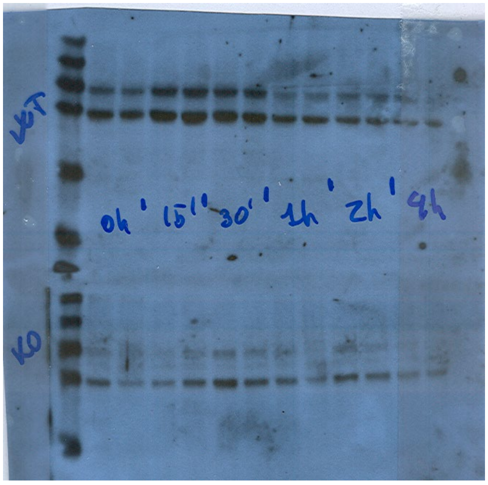

pJNK

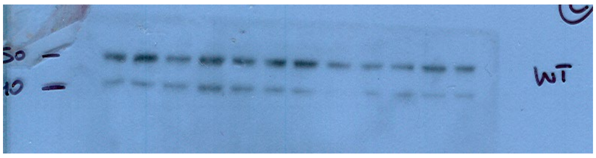

JNK

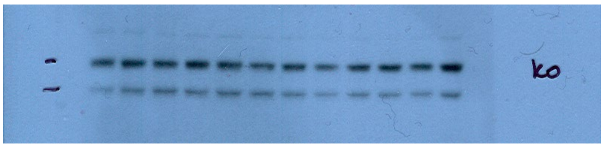

JNK

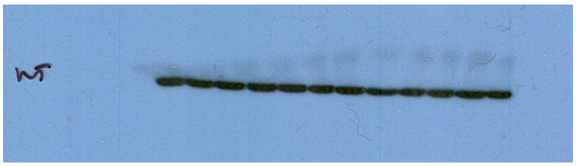

β Actin

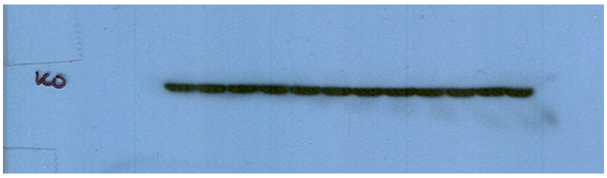

β Actin

Figure 5D

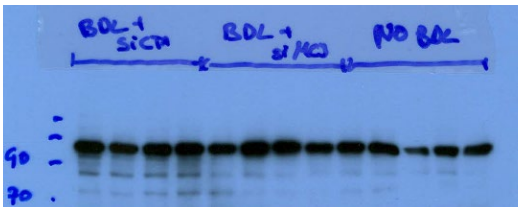

PARP

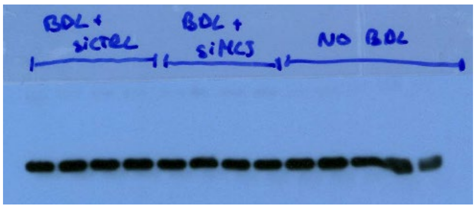

GAPDH
