# Supplementary material for: Boosting mitochondria activity by silencing MCJ overcomes cholestasis-induced liver injury
Source: JHEP Rep. 2021 Mar 18;3(3):100276. doi: 10.1016/j.jhepr.2021.100276 (PMC8099785; doi:10.1016/j.jhepr.2021.100276)
Supplement: Supplementary file 2 [file mmc2.pdf]

JHEP Reports

CTAT methods

Tables for a "Complete, Transparent, Accurate and Timely account" (CTAT) are now mandatory for all revised submissions. The aim is to enhance the reproducibility of methods.

- Only include the parts relevant to your study
- Refer to the CTAT in the main text as 'Supplementary CTAT Table'
- Do not add subheadings
- Add as many rows as needed to include all information
- Only include one item per row

If the CTAT form is not relevant to your study, please outline the reasons why:

1.1 Antibodies

Name	Citation	Supplier	Cat no.	Clone no.
Anti-mouse IgG, HRP-linked		Cell Signaling	7076	Polyclonal
Anti-rabbit IgG, HRP-linked		Cell Signaling	7074	Polyclonal
β-actina		Sigma-Aldrich	A5441	AC-15
GAPDH		Abcam	Ab8245	6C5
JNK		Cell Signaling	9252S	Polyclonal
MCJ		Dra. Rincon		WeNA12
PARP		Cell Signaling	9542	Polyclonal
pJNK (Thr183/Tyr185)		Invitrogen	4468G	Polyclonal
Tubulin		Sigma-Aldrich	T9026	DM1A
BAX		Cell Signaling	2772S	
BCL-Xl		Santa Cruz	sc-7195	
MLKL		Millipore Sigma	SAB1302339	
pMLKL		Sigma-Aldrich	T9026	

1.2 Cell lines

Name	Citation	Supplier	Cat no.	Passage no.	Authentication test method

1.3 Organisms

Name	Citation	Supplier	Strain	Sex	Age	Overall n number
WT mice		CIC bioGUNE Animal Facility	C57BL/6J	Male	3 months	31

MCJ-KO mice		CIC bioGUNE Animal Facility	C57BL/6J	Male	3 months	20

1.4 Sequence based reagents

Name	Sequence	Supplier
9S ribosomal RNA	5'-GACTCCGGAACAAACGTGAGG-3'<br>5'-CTTCATCTTGCCCTCGTCCA-3'	Sigma-Aldrich
C-X-C motif chemokine ligand 1	5'- GGTGTCCCCAAGTAACGGAG-3'<br>5'-TTGTCAGAAGCCAGCGTTCA -3'	Sigma-Aldrich
C-C motif chemokine ligand 2	5'- GACCCCAAGAAGGAATGGGT -3'<br>5'- ACCTTAGGGCAGATGCAGTT -3'	Sigma-Aldrich
C-C motif chemokine receptor 2	5'- ATCCACGGCATACTATCAACAT -3'<br>5'- CAAGGCTCACCATCATCGTAG-3'	Sigma-Aldrich
C-C motif chemokine receptor 5	5'- GTGTGGAAAATGAGGACTGCAT -3'<br>5'- GTCAGAACGGTCAACTTTGGG -3'	Sigma-Aldrich
Enhancer of zeste homolog 2	5'- GGGAGCAAAGCTTGCATTCAT-3'<br>5'-GGAAGGGATGTAGGAAGCAGTC-3'	Sigma-Aldrich
Glyceraldehyde-3-phosphate dehydrogenase	5'-CGTCCCGTAGACAAAATGG-3'<br>5'-TTGATGGCAACAATCTCCAC-3'	Sigma-Aldrich
Hypoxia inducible factor 1 subunit alpha	5'-TCAAGTCAGCAACGTGGAAG-3'<br>5'-TATCGAGGCTGTGTCGACTG-3'	Sigma-Aldrich
Interleukin 1β	5'-GCCACCTTTTGACAGTGATGAG-3'<br>5'GACAGCCCAGGTCAAAGGTT-3'	Sigma-Aldrich
Interleukin 10	5'-GGTTGCCAAGCCTTATCGGA-3'<br>5'-ACCTGCTCCACTGCCTTGCT-3'	Sigma-Aldrich
Methylation J-Controlled	5' -ACGCCGACATCGACCACACAG-3'<br>5'-AATCTTCCTTGCTGTTGCCGTC-3'	Sigma-Aldrich
Nuclear receptor factor 1	5'-CTTCATGGAGGAGCACGGAG-3'<br>5'-CGTGGAGTTGAGGATGTCCC-3'	Sigma-Aldrich
Peroxisome proliferator-activated receptor gamma coactivator 1-alpha	5'-AGACAGGTGCCTTCAGTTCAC-3'<br>5'-ACCAGAGCAGCACACTCTATG-3'	Sigma-Aldrich
Peroxisome proliferator-activated receptor gamma coactivator 1-beta	5'-TCTGACGTGGACGAGCTTTC-3'<br>5'-CGTCCTTCAGAGCGTCAGAG-3'	Sigma-Aldrich
Transcription factor A, mitochondrial	5'-CTGCCTTCCTCTAGCCCGGG-3'<br>5'-GTAACAGCAGACAACTTGTG-3'	Sigma-Aldrich
Tumor necrosis factor	5'- CGTCAGCCGATTTGCTATCT-3'<br>5'-CGGACTCCGCAAAGTCTAAG -3'	Sigma-Aldrich
TNF-related apoptosis inducing ligand	5'- CCAACGAGATGAAGCAGC-3'<br>3'- CCATCAGTGGAGTCCCAG-3'	Sigma-Aldrich
Uncoupling protein 2	5'-AAAGCAGCCTCCAGAACTCCG-3'<br>5'-TGGAGAAACGGGGACCTTCA-3'	Sigma-Aldrich

1.5 Biological samples

Description	Source	Identifier
Human liver samples	Marqués de Valdecilla Hospital<br>Pomeranian Medical University	2017-052
Human serum samples	Marqués de Valdecilla Hospital<br>Pomeranian Medical University<br>Puerta de Hierro Hospital<br>Virgen de Victoria Hosìtal	2017-052

1.6 Deposited data

Name of repository	Identifier	Link

1.7 Software

Software name	Manufacturer	Version
FRIDA software	Johns Hopkins University	
ImageJ	http://rsbweb.nih.gov/ij	
SPSS Statistics softwate	IBM	19.0

1.8 Other (*e.g*. drugs, proteins, vectors etc.)

MCJ-Specific siRNA	Ambion	Cat# 4457307
pcDNA3-LacZ	Invitrogen	
Hematoxylin solution	Sigma	
Eosin solution	Sigma	
Sirius red picric acid solution	Panreac	
Trizol reagent	Invitrogen	
DNAse	Invitrogen	
DuoSet II kit	R&D Systems	
Glycochenodeoxycholic acid	Sigma	
Myeloperoxidase Colorimetric Activity Assay Kit	Sigma-Aldrich	
M-MLV Reverse Transcriptase	Invitrogen	
SYBR Select Master Mix	Applied Biosystems	
Succinate Dehydrogenase Activity Colorimetric Assay Kit	Sigma-Aldrich	
Jetprime reagent	Polyplus	
Total Bile Acid Assay Kit	Cell Biolabs	
Deoxycholic acid	Sigma	
Fluorogenic caspase-3 substrate	Enzo Life Sciences	

JC-1 dye	Life Technologies	
MitoSOX Red mitochondrial superoxide	Life Technologies	
ATPlite luminescence ATP detection assay system	Perkin Elmer	

1.9 Please provide the details of the corresponding methods author for the manuscript:

María Luz Martínez-Chantar, CIC bioGUNE, Bizkaia Science and Technology Park, Derio 48160, Bizkaia, Spain; email: mlmartinez@cicbiogune.es

2.0 Please confirm for randomised controlled trials all versions of the clinical protocol are included in the submission. These will be published online as supplementary information.
